# Computer-Aided System of the Mandibular Cortical Bone Porosity Assessment on Digital Panoramic Radiographs

**DOI:** 10.1055/s-0042-1749158

**Published:** 2022-09-19

**Authors:** Eha R. Astuti, Agus Z. Arifin, Rarasmaya Indraswari, Ramadhan H. Putra, Nastiti F. Ramadhani, Berty Pramatika

**Affiliations:** 1 Department of Dentomaxillofacial Radiology, Faculty of Dental Medicine, Airlangga University, Surabaya, Indonesia; 2 Department of Informatics, Faculty of Intelligent Electrical and Information Technology, Institut Teknologi Sepuluh Nopember, Surabaya, Indonesia; 3 Department of Information Systems, Faculty of Intelligent Electrical and Information Technology, Institut Teknologi Sepuluh Nopember, Surabaya, Indonesia; 4 Graduate Student of Dental Health Sciene Program, Faculty of Dental Medicine, Airlangga University, Surabaya, Indonesia; 5 Universitas Brawijaya Hospital, Brawijaya University, Malang, Indonesia

**Keywords:** computer-aided system, dental panoramic radiographs, mandibular cortical bone, osteoporosis, bone mineral density

## Abstract

**Objectives** The loss of bone mineral density (BMD) in various sites of the body, including the mandible, is the main sign of osteoporosis. Thus, the computer-aided diagnosis (CAD) system was developed for bone density assessment and patients were classified into normal, osteopenia, and osteoporosis groups using a digital panoramic radiograph.

**Material and Methods** Data of dental panoramic radiographs and corresponding BMD assessments from 123 postmenopausal women were collected. For the proposed CAD system test, regions of interest (ROI) that were located below the left and right mental foramen on dental panoramic radiographs were determined. The width and texture of the mandibular cortical bone in each ROI were used to classify the data into normal, osteopenia, and osteoporosis classes. The width of the mandibular cortical was measured using the polynomial fitting method. The texture feature of the cortical bone is obtained by calculating the average value of the grayscale intensity of cortical bone. The classification result was obtained by using a multiclass support vector machine.

**Results** The experimental results using 10-fold cross-validation showed that the proposed system achieved an average accuracy of 86.50% for osteoporosis classification on dental panoramic radiographs. The average misclassification error and relative foreground area error of the segmentation process were 5.21 and 12.98%, respectively. From the analysis of the cortical width measurement process, highest average mandibular cortical width (MCW) was found in the normal patient category compared with the other classes.

**Conclusion** This research showed that the proposed computer-aided system can be used for osteoporosis and osteopenia assessment by measuring the MCW and texture on dental panoramic radiographs with the average system accuracy of 89.52%.

## Introduction

The continuous loss of bone tissue and bone mineral density (BMD) in the skeletal system is a sign of osteoporosis. 1 Osteoporosis is referred to as a “silent disease” because it does not cause obvious symptoms and is usually detected when the bone fracture has already presented. 2 BMD measurement using a dual energy X-ray absorptiometry (DEXA) device is the gold standard for diagnosing osteoporosis by assessing the condition of the bones. Since low BMD can affect any skeletal bone, osteoporosis can also manifest in the mandible. 3 Many studies have reported that digital panoramic radiographs can be used as a tool to assist in the detection of osteoporosis by measuring and analyzing the cortical bone width.

Early detection of osteoporosis is essential to prevent more damage. 2 This prevention can be done by detecting the early stage of bone loss by low BMD, which is commonly referred to as osteopenia. Women with mild osteopenia can develop osteoporosis by 15 years, while women with advanced osteopenia can develop osteoporosis within 1 year. 4 Therefore, lifestyle modification in osteopenia can be done to prevent bone mass and structural deterioration leading to osteoporosis. Although osteopenia has a lower risk of fracture compared with osteoporosis, its detection becomes more important because the prevalence of fracture in postmenopausal women with osteopenia is 21%. Moreover, the number of persons with osteopenia is much higher than the number of persons with osteoporosis. Therefore, a substantial proportion of the population is at risk of fracture. 5

Digital panoramic radiographs have been used in several radiomorphometric studies to predict osteoporosis based on the BMD level. Mandibular cortical width (MCW), panoramic mandibular index, and Klemetti index are overall useful tools that potentially could be used by dentists to screen for low BMD and predict osteoporosis. According to the meta-analysis, the erosion of mandibular cortical bone gives an estimated sensitivity and specificity in detecting osteoporosis, respectively, of 0.806 and 0.643. Additionally, MCW also shows a better accuracy in excluding osteopenia/osteoporosis (specificity) because patients with cortical width more than 4 mm have a normal BMD in 90% of the cases. 6 Since the panoramic radiograph is commonly used as a diagnostic tool in dental practice, it will be very useful for the initial screening of the osteoporosis risk and recommending the examination of additional clinical risk factors to prevent osteoporotic fracture.

However, manually measuring the potential parameters in a panoramic radiograph is time-consuming and prone to measurement errors. To assist clinicians, automated osteoporosis detection using digital panoramic radiographs is generally performed by measuring the width of cortical bone at the mandible. A computer-aided system for identifying osteoporosis using a digital panoramic radiograph revealed that the correlation between cortical bone width and BMD measurement result is approximately 53%. 7 In this accord, the study by Geary et al 8 also demonstrated a significant correlation between the width of cortical bone and bone density, but the significance level decreased when combined with other factors, such as age, length and width of the jaw, and the number of teeth, to detect bone density.

Several studies have been conducted to detect osteoporosis by measuring the bone density of the mandible using cone-beam computed tomography (CBCT) imaging. CBCT is being widely used in dentistry because it can provide better imaging quality with less radiation dose compared with CT scans. 9 Alkhader et al 9 measured the bone density of mandibular condyle (BDMC) of 204 patients who were at the risk of osteoporosis. The result of this study was that the BDMC can be useful for predicting osteoporosis. Alkhader et al 10 also conducted a study that measured the radiographic density of the axis vertebra (RDAV) on the CBCT of 247 patients who were at the risk of osteoporosis, which showed that RDAV can also be used for predicting osteoporosis. Even though CBCT can provide better imaging quality than digital panoramic radiographs, the CBCT examination costs more than the panoramic radiograph examination. Therefore, CBCT imaging may not be suitable for early detection purposes. Moreover, CBCT imaging delivers a higher radiation dose compared with panoramic radiographs. 11 12

In this study, we developed a computer-aided diagnosis (CAD) system to assist osteoporosis assessment based not only on MCW but also on analysis of the texture of the cortical bone representing the bone porosity. This assessment was performed using digital panoramic radiographs and patients were classified into normal, osteopenia, or osteoporosis groups using multiclass classification with the support vector machine (SVM). To validate the proposed CAD system, we compared the classification result of the CAD system to the BMD measurement of the patient using the DEXA device. The result of this CAD system can be used for the early detection of osteoporosis disease.

## Materials and Method

The research subjects were postmenopausal women who underwent lumbar spine DEXA and panoramic radiograph examination in our clinic from January 2019 to July 2020. The total sample for our research was 123 postmenopausal women. The study inclusion criteria were women who had not menstruated for at least 1 year. The study exclusion criteria were subjects with metabolic bone disease; cancers with bone metastasis; subjects taking medications that affect bone metabolism, such as estrogen; subjects with a smoking history; and subjects with lesions in the mandible.

The panoramic radiographs of all subjects were obtained using the Instrumentarium OP 200D-1 Digital Panoramic and Cephalometric System (70 kVp, 8 mA, 12s). This study protocol was approved by the Ethics Committee Medical Faculty, Airlangga University, Surabaya (603 / HRECC. FODM / IX / 2019). Two dental radiologists examined all of the panoramic radiographs. Both observers determined regions of interest (ROI) on panoramic radiographs for the CAD system test.

We classified all of the 123 patients into three groups based on BMD measurement by DXA examination. A total of 68, 38, and 17 patients were classified into normal, osteopenia, and osteoporosis groups, respectively. According to the World Health Organization criteria, a patient is diagnosed with osteoporosis when a T value of 2.5 SD or more below the young female adult mean is recorded, while a T score between –1 and –2.5 SD defines status of osteopenia.

**Fig. 1:**
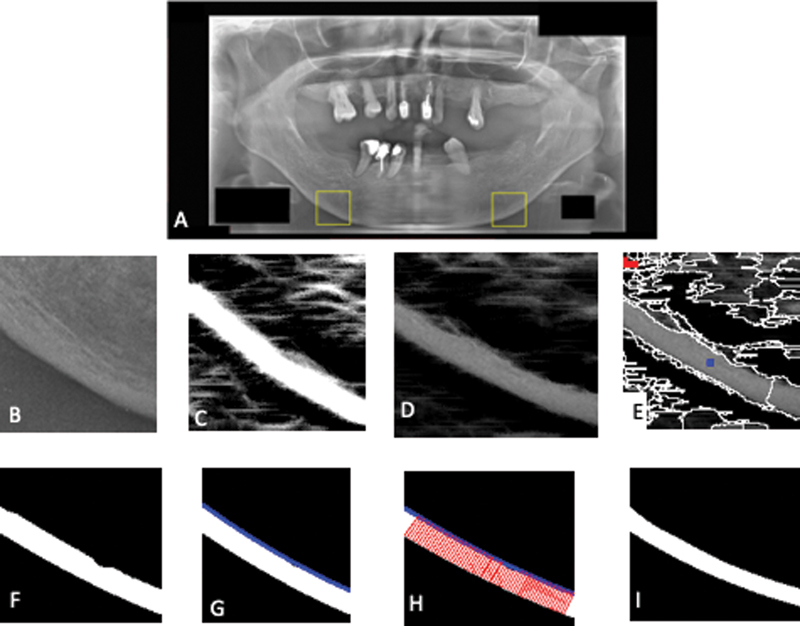
The process of proposed CAD system: ( **A** ) dental panoramic radiographs and the selected ROIs; ( **B** ) cropped ROIs; ( **C** ) multiscale line operator result; ( **D** ) multiplication result; ( **E** ) initial segmentation an automatic marking; ( **F** ) segmentation result; ( **G** ) polynomial function; ( **H** ) line tangent; and ( **I** ) ground truth. CAD, computer-aided diagnosis; ROI, region of interest

The proposed system consisted of four main processes, which were the selection of ROI, cortical bone segmentation, extraction of width and texture features from cortical bone, and classification. The result of each step of the proposed system is shown in Fig. 1 . The proposed system measured the cortical bone width and texture to predict low BMD based on the existing indexes (such as MCW, panoramic mandibular index, and Klemetti index), which showed that the mandibular cortical bone width and porosity were related to the BMD. The proposed CAD system was developed using Matlab R2013a software.

## Region of Interest

The ROI used in this research is the cortical bone that is located at the bottom of the mental foramen because this area is generally used as a reference for radiomorphometric analysis to detect osteoporosis using a panoramic radiograph. 6 Two ROIs from left and right sides of the jaw were cropped into the size of 128 × 128 pixels.

## Cortical Bone Segmentation

In this research, we proposed the integration between multiscale line operators for preprocessing with statistical region merging for the segmentation process. Before the operator performed the segmentation process, preprocessing with the multiscale line was done to clarify the structure of cortical bone.

## Multiscale Line Operator

The multiscale line operator is commonly used for detecting linear structures in fibers, retinal vessels, mammograms, and trabecular bone in dental panoramic radiographs. 13 14 15 16 The first step of the multiscale line operator is performing multiscale analysis using a Gaussian image pyramid to generate images on multiple scales. 17 The second step is calculating the line strength of each pixel in the image that is generated by the Gaussian image pyramid using the line operator. To preserve the gray value information of the initial ROI, multiplication of the initial ROI with the multiscale line operator result was used as the input of the next process. This multiplication image can reinforce the bone structure of the cortical bone in the ROI image while maintaining the important gray value information of the ROI image.

## Statistical Region Merging

It was still difficult to separate the cortical bone from the trabecular bone structure using the multiscale operator method. Therefore, we continued with the statistical method of merging region. This statistical region merging method is a semiautomatic segmentation because it requires input from the user to do the segmentation process. There are four main processes in statistical region merging, which are initial segmentation, sample area selection, distance measurement between regions, and region merging.

To determine the sample area of the object class, we select several large and brightly colored areas, called the object candidates. From the object candidates, we select an area that is located in the middle of the image as the sample area of the object class. Then, the background class is determined as a small area that is located far from the middle of the image and has the smallest gray intensity. Fig. 1E shows the result of the automatic sample region selection where the sample area of the object class is marked with a blue dot while the sample area of the background class is marked with a red dot.

The researchers measured the distance between areas that have not been marked with the sample area. Our research used the distance measurement and region merging method proposed by Arifin et al. 18 The area that has the smallest distance will be merged into the object or background class. This process is done iteratively until the entire area of the image is merged into either object or background class.

## Features Extraction

The feature extraction process includes an extraction process of the width and texture of cortical bone, which is used to classify osteoporosis. The width and texture features of cortical bone are used to assess the level of bone loss, in which porous bones becomes thinner and appears hollow compared with nonporous bones.

## Cortical Bone Width Measurement

The researchers used the polynomial fitting method (a method to determine the upper border of cortical bone) to obtain the width of cortical bone by matching the process of polynomial function line with the upper border of the cortical bone. Polynomial functions that give the least square error were selected as the function that mostly represented the upper border of the cortical bone.

At each point on the polynomial function curve, cortical bone width was measured continuously. Line tangent calculation was used to determine the upper edge point corresponding to the direction of the polynomial curve. Then, a line that was perpendicular to the line tangent was made to the point on the bottom border of the cortical bone. The length of the line was the width or thickness of the cortical bone.

## Cortical Bone Texture Analysis

First, we did normalization of the grayscale intensity of the dental panoramic radiograph's ROIs in Fig. 1B , so that each image could have the same intensity. We needed this step because there was uneven illumination in the panoramic image of the tooth so that each ROI could have a different intensity value of the “dark” (porous) area. The average value of the grayscale intensity on the normalized image was calculated. The average value of grayscale intensity on the right and left ROIs resulting from the normalization process was the result of feature extraction of cortical bone texture.

## Classification

The researcher utilized eight features for the classification process, such as the minimum, maximum, and average widths of left and right cortical bones, and the average grayscale value of the right and left cortical bones. The multiclass SVM was used to classify the input data into normal, osteopenia, and osteoporosis classes. 19 We used radial basis functions in the SVM classification.

## Data Analysis

In this research, several experiments were conducted to calculate the accuracy of the proposed system, the errors of the proposed segmentation method, and to analyze the cortical bone width measurement results. To calculate the system accuracy, the ground truth of the normal, osteopenia, and osteoporosis class labels for each patient obtained from BMD measurement of the patients' spines was used. To calculate the error of the segmentation method, the manual segmentation of cortical bones of each dental panoramic radiograph by the experts was used as the ground truth ( Fig. 1I ).

## Results

### Calculation of System Accuracy and the Effect of Texture Feature

All digital panoramic radiographs on the dataset were divided into training data and test data. System accuracy was calculated by calculating the percentage of test data successfully classified by the system. To validate the accuracy of the system, the *k* -fold cross-validation method was used where the data were divided into *k* -folds randomly and equally. The *k* with the value of 2, 5, and 10 were used. One-fold was used as test data, and its accuracy was calculated while the remaining folds were used as training data. The process was done alternately to the entire folds. The obtained accuracy from each fold was averaged to obtain the accuracy of the system. In addition, a comparison of system accuracy when using only the cortical bone width feature was done to assess whether the cortical bone texture feature has an influence on the accuracy of the system.

Table 1 shows that the highest accuracy of the osteoporosis detection system is 86.50% and obtained using parameter *k* = 10. If the system only used the cortical bone width feature, the obtained system accuracy was always lower compared with when both width and texture features were used. The highest accuracy of the system was 74.04% when only using the cortical bone width feature, which obtained at the value of *k* = 10. From this experiment, better performance was observed when both features of cortical width and texture were used to detect osteoporosis because it had a higher accuracy value compared with only single feature of cortical bone width.

**Table 1:** Accuracy of osteoporosis detection system

***k***	**Using width and texture features (%)**	**Using width features (%)**	**Using texture features (%)**
2	86.14	73.96	46.36
3	86.20	73.87	46.52
5	86.24	73.73	46.49
10	86.50	74.94	46.77
Average	86.27	73.90	46.54

### Calculation of Segmentation Error

**Fig. 2:**
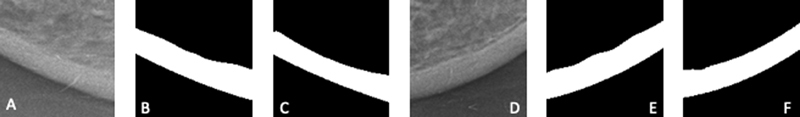
Segmentation result on ROIs in normal class: ( **A** ) right ROI; ( **B** ) ground truth of right ROI; ( **C** ) segmentation result of right ROI; ( **D** ) left ROI; ( **E** ) ground truth of left ROI; and ( **F** ) segmentation result of left ROI. ROI, region of interest.

**Fig. 3:**
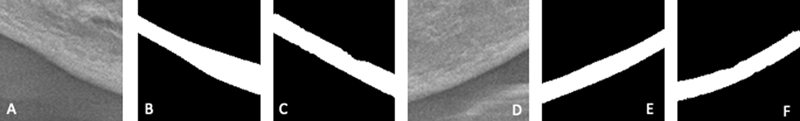
Segmentation result on ROIs in osteopenia class: ( **A** ) right ROI; ( **B** ) ground truth of right ROI; ( **C** ) segmentation result of right ROI; ( **D** ) left ROI; ( **E** ) ground truth of left ROI; and ( **F** ) segmentation result of left ROI. ROI, region of interest.

**Fig. 4:**
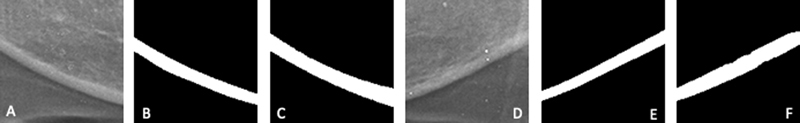
Segmentation result on ROIs in osteoporosis class: ( **A** ) right ROI; ( **B** ) ground truth of right ROI; ( **C** ) segmentation result of right ROI; ( **D** ) left ROI; ( **E** ) ground truth of left ROI; and ( **F** ) segmentation result of left ROI. ROI, region of interest.

The calculation of segmentation error was done by comparing the segmentation result and ground truth. Segmentation errors were calculated using misclassification error (ME) and relative foreground area error (RAE). RAE calculates the ratio of the object in the segmentation result with the object area in the ground truth, whereas ME calculates the percentage of pixels that are correctly classified as the object and the background. The example of segmentation results on a total of six ROI images from each class is shown in Figs. 2 to 4 . The average of ME and RAE values of 123 patients was 5.21 and 12.98%, respectively.

### Analysis of Features Extraction Process

Following six features of cortical bone width or thickness were taken: minimum width (min), maximum width (max), and average width (avg) of left and right cortical bone, and two textures features, which were the average intensity of right and left cortical bone. For the width features, the unit used was pixel. Table 2 shows the average value of the eight cortical bone features according to their actual class based on the BMD measurement using the DEXA device (normal, osteopenia, and osteoporosis).

**Table 2:** Average value of cortical bone features on each class (DEXA output)

**Class**	**ROI**	**Features**	***p* -Value **
Normal	Right	Minimum width	23.2 ± 6.6
		Maximum width	32.3 ± 6.6
		Average width	27.7 ± 6.4
		Texture	51.8 ± 11.9
	Left	Minimum width	21.8 ± 5.4
		Maximum width	31.2 ± 6.0
		Average width	26.6 ± 5.2
		Texture	47.1 ± 12.4
Osteopenia	Right	Minimum width	20.7 ± 5.2
		Maximum width	31.0 ± 7.0
		Average width	25.7 ± 5.6
		Texture	48.4 ± 11.0
	Left	Minimum width	19.0 ± 3.9
		Maximum width	30.8 ± 6.2
		Average width	24.9 ± 4.4
		Texture	45.5 ± 9.5
Osteoporosis	Right	Minimum width	19.0 ± 3.5
		Maximum width	26.5 ± 4.7
		Average width	22.9 ± 4.1
		Texture	44.9 ± 7.2
	Left	Minimum width	16.6 ± 3.1
		Maximum width	27.0 ± 6.1
		Average width	21.6 ± 4.4
		Texture	41.1 ± 10.2

Abbreviation: ROI, region of interest.

### CAD System Prediction

The assessment of the confusion matrix was done by two dental radiologist (E.R.A. and B.P.). The dental radiologist determined the ROI for the osteoporosis assessment in the system and manual osteoporosis assessment. Intraobserver agreement of the osteoporosis assessment by using CAD was 74 and 78% for the first and second dental radiologists, respectively. Interobserver agreement of the osteoporosis assessment by using CAD between the two radiologists was 81%.

Table 3 shows the CAD system prediction for the classification of all of the data in the dataset. The highest error was achieved when the system wrongly classified the normal data into osteoporosis class (eight data). The system also wrongly classified four data of normal class into osteopenia class. The system had no problem in classifying the osteoporosis data. The sensitivity, specificity, and accuracy of the system are presented in Table 4 .

**Table 3:** CAD system prediction in women with low bone density at 95% confidence level

**CAD system's prediction**					
**Identifying side**	**Sensitivity (95%CI)**	**Specificity (95%CI)**	**Positive prediction value (95% CI)**	**Negative prediction value (95% CI)**	**Accuracy (95%CI)**
Mandibular cortical inferior	98.21%	82.35%	5.57 (3.33–9.31)	0.02 (0.00–0.15)	89.52%
	(90.45–99.95%)	(71.20–90.53%)			(82.74–94.30%)

Abbreviations: CAD, computer-aided diagnosis; CI, confidence interval.

**Table 4:** CAD system prediction

		**System's prediction**			**Total actual class**
		**Normal**	**Osteopenia**	**Osteoporosis**	
Actual class	Normal	56	4	8	68
	Osteopenia	1	33	4	38
	Osteoporosis	0	0	17	17
Total predicted class		57	37	29	123

Abbreviation: CAD, computer-aided diagnosis.

## Discussion

Osteoporosis is a degenerative disease that can occur in both men and women. Early detection of osteopenia and osteoporosis is important to prevent the serious complications of osteoporosis, such as compression fractures or back pain. Although the DEXA scan is the gold standard for the osteoporosis assessment, the access of the DEXA-scan machine is limited in our city and the cost is expensive. Previous studies showed that there was a significant correlation between BMD on DEXA and mandibular cortical shape on panoramic radiographs in postmenopausal females. There were many studies demonstrating that panoramic radiographs could be used to assess the porosity of cortical bone. Therefore, dentists could detect low bone density based on panoramic radiograph assessment.

We proposed the CAD system to assess osteopenia and osteoporosis to reduce the subjectivity of the manual osteoporosis assessment. Our proposed system showed that the low bone density accuracy increased when we combined the calculation of bone width and bone texture features (89.2%). The accuracy was higher than the previous research. 7 20 The research result conducted by Arifin et al 7 showed that there was a statistically significant correlation between cortical width measured by the CAD system and spinal BMD. The accuracy of the CAD system for low BMD detection was 64 to 66%. Kavitha et al 21 demonstrated that the addition of mandibular cortical texture in cortical width texture improved the accuracy of osteoporosis detection with a total accuracy of 95.3 to 96.8%. In 2020, Nakamoto et al developed an osteoporosis diagnostic system for osteoporosis detection by using panoramic radiographs. Their study used cortical porosity of the mandibular bone to detect osteoporosis. The system showed high diagnostic efficacy with sensitivity of 90.9%, specificity of 64.7%, and accuracy of 75.0%. 20

In addition to the cortical bone width feature, a cortical bone texture feature that represented the cortical bone porosity was also considered as an important factor to predict low BMD. A study of bone mechanical properties and changes in osteoporosis by Osterhoff et al 22 revealed that the increasing number and size of porosity in the cortical bone can determine intracortical porosity and has association with the decreasing of BMD. Therefore, adding cortical bone texture features can increase the prediction performance of osteoporosis, rather than using only cortical bone width. The result of osteoporosis prediction might be increased when another potential feature in the digital panoramic radiograph is added.

Comparisons of our experimental results with those of previous studies demonstrated the feasibility performance of our proposed system in diagnosing high-risk groups with osteopenia or osteoporosis. However, there are some limitations of our CAD system. Inaccurate determination of ROI (posterior region of the foramen mentale) on a panoramic radiograph caused inaccurate bone classification. In addition, the os hyoid superimposition in the posterior part of the mandible contributed to errors in calculating cortical thickness and bone texture. Our study showed that there was an error classification of the dataset in the system; the possible reason was the sample of our study was varied based on the characteristic of edentulousness. The sample of our study consisted of the edentulous ridge women group and the total dentated. The mandibular bone morphology is influenced by genetic, systemic, and biomechanical functional factors. The biomechanical factors that influence bone density are the loss of the functional influence of teeth on the surrounding tissue, masticatory muscle function, direct loading of the alveolus by dentures, continuous wearing of ill-fitting dentures, and periodontal disease. The thicker and denser cortical layers of the mandible were an adaptive response to torsion and bending during mastication. 23 24 Another limitation of our study was the relatively small number of subjects, especially those with osteoporosis. As our study also needed to classify patients with osteopenia, a larger number of subjects is needed to acquire better results. Future well-designed studies with a larger number of subjects that also include more potential features of osteoporosis are needed to obtain higher accuracy, sensitivity, and specificity of osteoporosis prediction.

## Conclusion

In this research, a CAD system was proposed to measure width or thickness and analyze cortical bone texture in the mandibular cortical bone in panoramic dental images. This system can be used to assist osteopenia and osteoporosis detection, with average system accuracy of 89.52%. The average value of cortical bone features, both width and texture, is highest in patients in the normal class, followed by patients in the osteopenia and osteoporosis classes. The use of cortical bone width and texture features can provide a higher system accuracy value than using only the cortical bone width feature. Further studies compared with other types of radiographic imaging such as CBCT may be needed so that radiographic examination can be one of the methods for early detection of osteoporosis accurately.
